# Open-label phase 3 study of diclofenac conjugated to hyaluronate (diclofenac etalhyaluronate: ONO-5704/SI-613) for treatment of osteoarthritis: 1-year follow-up

**DOI:** 10.1186/s12891-021-04108-9

**Published:** 2021-03-01

**Authors:** Yoshihiro Nishida, Kazuyuki Kano, Taiki Osato, Takayuki Seo

**Affiliations:** 1 Department of Rehabilitation, Orthopaedic Surgery, Nagoya University Hospital, 65 Tsurumai-cho, Showa-ku, Nagoya, Aichi 466-8560 Japan; 2 Clinical Development Department, Research & Development Division, Seikagaku Corporation, Chiyoda-ku, Tokyo, Japan

**Keywords:** Osteoarthritis, Diclofenac etalhyaluronate, Phase 3 study, Intra-articular injection

## Abstract

**Background:**

We evaluated the 1-year safety and efficacy of diclofenac etalhyaluronate (DF-HA), a diclofenac-conjugated hyaluronate, in patients with osteoarthritis (OA).

**Methods:**

In this multi-centre, open-label, noncomparative phase 3 study in Japan, patients with a diagnosis of knee, shoulder, elbow, hip, or ankle OA received an intra-articular (IA) injection of DF-HA 30 mg every 4 weeks for 1 year (13 times in total). The safety outcomes included treatment-emergent adverse events (TEAEs) and target joint structural changes by X-ray imaging tests. Efficacy outcomes included joint pain scores on an 11-point numerical rating scale. Concomitant use of analgesics was not restricted.

**Results:**

Overall, 166 eligible patients were enrolled, comprising knee OA (*n* = 126) and other OA (*n* = 40). All TEAEs were experienced by 126/166 patients (75.9%). The incidence of treatment-related TEAEs was not associated with the treatment period. No significant worsening of joint status was observed in X-ray imaging tests at week 52 or at last assessment. The mean joint pain scores (± standard deviation) were 5.9 ± 1.2, 4.9 ± 1.9, and 3.1 ± 2.3 at baseline, and weeks 2 and 52, respectively. Improvement of pain score was observed after the first injection and was maintained until week 52 regardless of knee OA or other joint OA.

**Conclusions:**

Repeated IA injections of DF-HA every 4 weeks for 1 year were well tolerated with no clinically significant adverse events indicating they might lead to the long-term improvement of OA symptoms. DF-HA might be a useful treatment for patients with OA.

**Trial registration number:**

JapicCTI-183855 (First registered date: 6th February 2018).

## Background

Osteoarthritis (OA), the most common joint disorder, occurs frequently in the ageing population [1]. OA primarily affects the knee and hip, but can affect all joints. Major complaints of OA are joint pain, stiffness, and swelling leading to impaired health-related quality of life and activities of daily living [1]. OA can be managed by pharmacological and non-pharmacological therapies regardless of the affected joint. The pharmacological therapy approaches for OA commonly involve the oral or topical administration of nonsteroidal anti-inflammatory drugs (NSAIDs) or selective cyclooxygenase-2 inhibitors as well as intra-articular (IA) corticosteroids or hyaluronan (HA) for knee OA [2, 3].

Pharmacological treatment for OA requires a long duration because it is a symptomatic therapy that mainly relieves pain related to OA, which is a chronic disease. Long-term oral administration of NSAIDs increases the risk of gastrointestinal (GI) bleeding [4]. Because of the potential risk related to the long-term use of NSAIDs, it is recommended they are used at the lowest effective dose for the shortest possible time in patients with these risks [2, 3]. There is concern about the long-term use of IA corticosteroids because of the increased risk of accelerated OA progression or adverse joint events [5–7]. Furthermore, IA HA has a favourable safety profile and has been used to treat many patients with OA; however, the efficacy of IA HA for OA remains controversial [2, 3]. Medications available for long-term therapy with sufficient safety and efficacy are anticipated in patients with OA [5–7].

Diclofenac etalhyaluronate (DF-HA: ONO-5704/SI-613) is a novel HA derivative consisting of a diclofenac (DF) molecule covalently linked to HA and was developed as an IA-injectable drug for the treatment of OA [8]. Once DF-HA is injected into the articular cavity of a joint with OA, it gradually releases DF in a sustained manner, which exhibits anti-inflammatory effects lasting up to 28 days, suppression of joint cartilage degeneration and normalization of synovial fluid function [8, 9].

The efficacy and safety of IA injections of DF-HA 30 mg were evaluated in patients with knee OA in a randomised placebo-controlled phase 2 study [10] and a confirmatory phase 3 study (unpublished results). In these studies, DF-HA was injected up to six times every 4 weeks during a 6-month follow-up period, the use of analgesics was limited, and only the knee joint was targeted. Further safety and efficacy evidence for DF-HA is required for its clinical use in OA.

Therefore, we conducted this study (JapicCTI-183,855) to evaluate the safety and efficacy of repeated IA injections of DF-HA 30 mg (every 4 weeks) for 1 year in patients with OA.

## Methods

### Study design and patients

This was a multi-centre, open-label, noncomparative phase 3 study to evaluate the safety (primary objective) and efficacy of multiple doses of DF-HA (Seikagaku Corporation, Tokyo, Japan) injected over 1 year in patients with OA including knee-, shoulder-, elbow-, hip-, or ankle-joint OA in Japan, in accordance with the good clinical practices of the International Conference on Harmonization (ICH) guidelines. A Central Institutional Review Board (C-IRB) approved the study. All investigators are orthopaedic surgeons. Patients provided written informed consent after receiving the consent explanation document approved by the C-IRB. At screening, eligible patients were aged at least 20 years, with a diagnosis of OA by X-ray imaging regardless of OA grade or stage, with pain for at least 12 weeks, and an 11-point numerical rating scale (NRS) of 4–9 at the target joint. Key exclusion criteria were as follows: invasive procedure to the target joint including arthroscopy within 1 year prior to the start date of screening (except for removal of joint effusion), and radiographically-confirmed serious joint deformation such as osteophyte formation in the target joint which could require surgical intervention during a long-term study.

### Intervention

Screening was performed within 1 week prior to initial injection (week 0). After the screening, eligible patients received IA injection of DF-HA (a prefilled syringe containing DF-HA 30 mg/3 mL) every 4 weeks for 1 year (13 times in total). To administer DF-HA to the target joint accurately, DF-HA was injected by investigators with experience of IA injection following a manual prepared for the study, which defined the methods and approach to the articular cavity of each target joint. Ultrasound- or X-ray-guided injection was recommended for joints other than the knee joint, which has a large cavity and is usually injected blindly (without imaging assistance or guidance) in normal clinical practice in Japan.

Concomitant use of analgesics was not limited because some patients use DF-HA in combination with analgesics in clinical practice. Injection of other IA products except for HA at the same target joint was prohibited. However, the use of these medications for the target joint was permitted in cases of treatment for adverse events.

For assessment, patients visited their study centres at weeks 0, 2, 4, and every 4 weeks thereafter up to week 52.

### Outcomes

Safety outcomes were evaluated by treatment-emergent adverse events (TEAEs), clinical laboratory tests, vital signs, target joint examination, and X-ray imaging tests to observe structural worsening in the target joint. Causal relationships between TEAEs and DF-HA were judged by the investigators. TEAEs that led to study drug withdrawal were considered significant TEAEs, while those occurring at the injection site or associated with GI disorders, were considered TEAEs of special interest. Similarly, anaphylactic reaction or hypersensitivity were considered TEAEs of special interest because treatment-related anaphylactic reaction occurred in the confirmatory phase 3 study (2/220 patients) (unpublished results). Regarding the target joint examination, investigators assessed the joint condition (effusion, swelling, redness, and warmth) on three levels to judge whether the TEAE occurred when the target joint condition had worsened from the screening period. Investigators performed X-ray imaging with reference to the “X-ray imaging procedure manual” prepared separately from the protocol. Each joint X-ray image was captured in the anteroposterior and lateral views. For lower limb joints, X-rays were performed with the patient in the standing position.

Investigators structurally evaluated the worsening of target joint status (osteophyte, joint space narrowing, osteosclerosis, or deformity of epiphysis) by comparing the 24-week and 52-week X-ray images to the baseline images. Evaluation was based on criteria in the “Radiographic Assessment Manual” prepared separately from the protocol for unified evaluation.

Efficacy of DF-HA was assessed by pain scores using the 11-point NRS, patient/physician global assessment by 100-mm visual analogue scale (VAS), quality of life score by Medical Outcomes Study Short-Form 36-Item Health Survey (SF-36), EuroQol 5 dimension (EQ-5D) [11–14], ratio of improved patients, and use of analgesics for the treatment of OA. Joint specific questionnaires were conducted including the Western Ontario and McMaster University Osteoarthritis 3.1 index (WOMAC), Shoulder 36, Patient-Rated Elbow Evaluation of the Japanese Version (PREE-J), and Self-Administered Foot Evaluation Questionnaire (SAFE-Q) for knee/hip, shoulder, elbow, and ankle OA, respectively [15–19]. Because the purpose of this study was the evaluation of the safety of DF-HA, a primary efficacy endpoint was not set.

### Statistics

In accordance with ICH-E1 guidelines, the sample size for knee OA was set at 120 patients to detect TEAEs that occurred with an incidence of ≥3% at 95% power and considering the study’s discontinuation rate. The sample size for the study of OA of the shoulder-, elbow-, hip-, and ankle-joint was set at 40 patients, one-third of the knee OA sample size. Statistical analyses were conducted on knee OA and other OA as well as on total OA using SAS version 9.4 (SAS Institute, Cary, NC, USA).

Safety was evaluated in the safety set, which included patients who received treatment at least once. TEAEs were coded using the Medical Dictionary for Regulatory Activities (MedDRA) ver.22.0 and their incidences were calculated. TEAEs were also calculated by the timing of onset. TEAEs associated with GI disorders, anaphylactic reaction, and hypersensitivity were categorized by standardised MedDRA query (broad search). Safety outcomes were summarised in combination with or without systemic NSAIDs such as oral and suppository NSAIDs, and injected amount of HA to assess the safety when DF-HA was used concomitantly with other medications. For the safety evaluation of the amount of IA injected HA concomitantly with DF-HA, subgroup analysis of TEAEs was performed for three groups of patients: those who did not use HA; those who received it concomitantly for ≤24 weeks; and those for > 24 weeks, where 24 weeks corresponded to half of the planned treatment period of DF-HA for 1 year.

Efficacy was evaluated in the full analysis set (FAS), which included all patients who had at least one evaluation for efficacy after the first injection of DF-HA. Summary statistics of efficacy data were analysed. Patients with pain scores improved by ≥30% from baseline were defined as “responder”. For joint pain scores, subgroup analysis was performed in groups stratified according to with or without use of analgesics for OA of the target joint.

## Results

### Patients

**Fig. 1:**
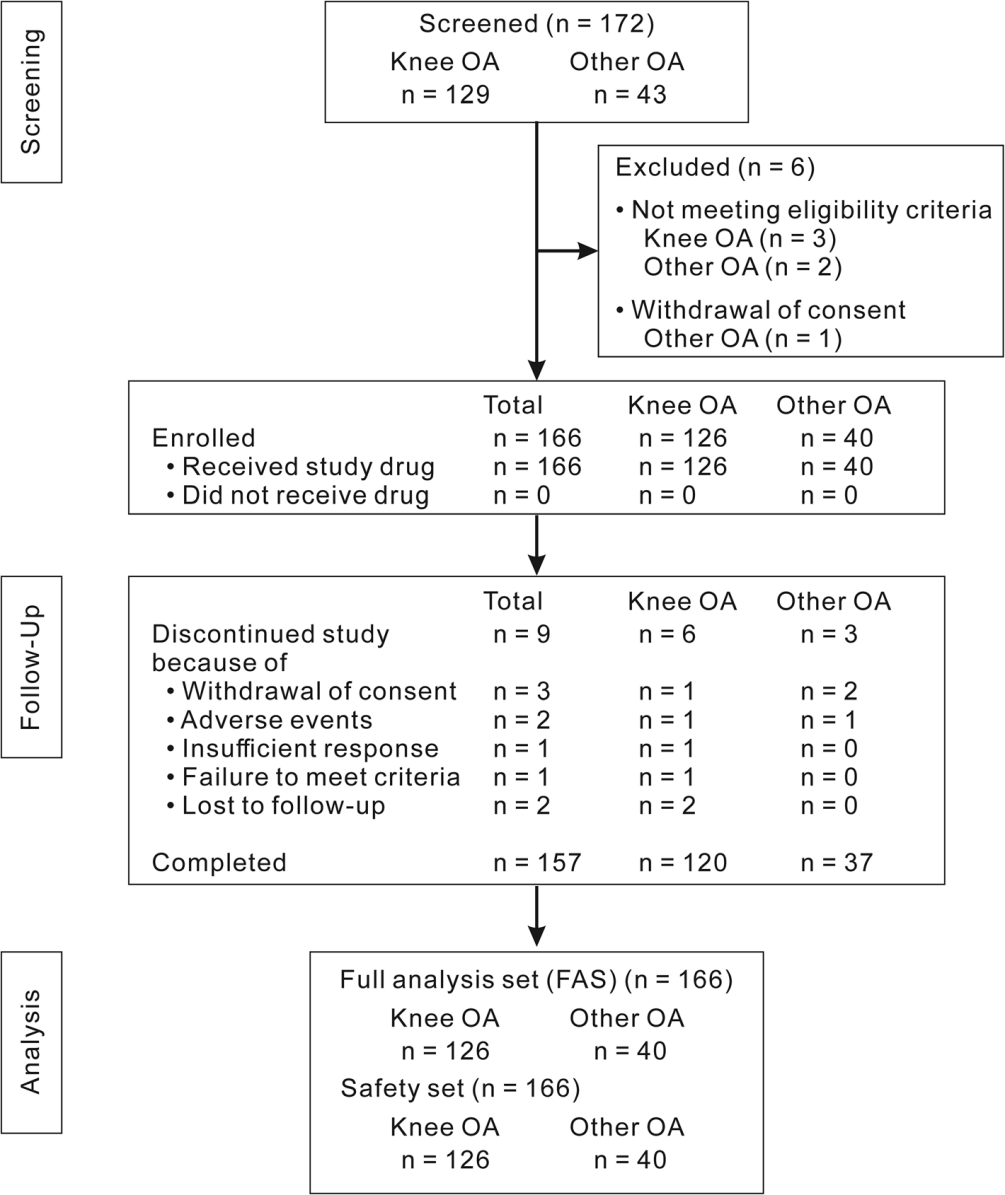
Patient disposition. OA = osteoarthritis

Between February 2018 and August 2019, 172 patients were screened for eligibility from 10 sites and 166 patients were enrolled comprising knee OA (*n* = 126), shoulder OA (*n* = 15), elbow OA (*n* = 8), hip OA (*n* = 9), and ankle OA (n = 8) (Fig. 1). All data from 166 enrolled patients were included in the FAS and safety set and 157 patients completed the study (Fig. 1). Most patients received a 3-mL formulation containing 30 mg of DF-HA, but 12 patients did not receive the whole amount of DF-HA at least once: knee (*n* = 7), elbow (*n* = 3), and ankle (*n* = 2). The major reasons for dosing volume reduction were injection pain in the joint. For some cases that experienced joint pain, the volume was reduced the next time to prevent pain.

The mean age was 65.9 years and women accounted for 69.9% (116/166) of patients. More women had knee OA compared with other OA. In terms of OA classification, primary OA accounted for 91.6% (152/166 patients) and secondary OA for 8.4% (14/166 patients) of patients (Table 1).

**Table 1:** Demographic and other baseline characteristics in the full analysis set

Characteristics		Knee joint *N* = 126	Other joint *N* = 40	Total *N* = 166
Age (years), n (%)	Mean ± SD	67.0 ± 10.2	62.1 ± 13.4	65.9 ± 11.2
	< 65	51 (40.5)	19 (47.5)	70 (42.2)
	≥65–75	45 (35.7)	14 (35.0)	59 (35.5)
	> 75	30 (23.8)	7 (17.5)	37 (22.3)
Sex, n (%)	Male	33 (26.2)	17 (42.5)	50 (30.1)
	Female	93 (73.8)	23 (57.5)	116 (69.9)
BMI (kg/m^2^), n (%)	Mean ± SD	26.06 ± 4.92	25.33 ± 4.49	25.88 ± 4.82
	< 25.0	54 (42.9)	20 (50.0)	74 (44.6)
	≥25.0	72 (57.1)	20 (50.0)	92 (55.4)
Duration of current joint pain (years), n (%)	Mean ± SD	5.7 ± 6.4	4.1 ± 5.3	5.3 ± 6.2
	< 1	31 (24.6)	13 (32.5)	44 (26.5)
	≥1	95 (75.4)	27 (67.5)	122 (73.5)
Classification of OA, n (%)	Primary	122 (96.8)	30 (75.0)	152 (91.6)
	Secondary	4 (3.2)	10 (25.0)	14 (8.4)
Cause^a^, n (%)^b^	Injury	2 (50.0)	1 (10.0)	3 (21.4)
	Cuff tear		2 (20.0)	2 (14.3)
	Meniscus injury	2 (50.0)		2 (14.3)
	Acetabular dysplasia		6 (60.0)	6 (42.9)
	Other	0	1 (10.0)	1 (7.1)
Stage of OA^c^				
Knee, shoulder and elbow OA, n (%)	Grade 1	18 (14.3)	13 (32.5)	31 (18.7)
	Grade 2	52 (41.3)	5 (12.5)	57 (34.3)
	Grade 3	43 (34.1)	4 (10.0)	47 (28.3)
	Grade 4	13 (10.3)	1 (2.5)	14 (8.4)
Hip OA, n (%)	Early stage		5 (12.5)	5 (3.0)
	Advanced stage		3 (7.5)	3 (1.8)
	End stage		1 (2.5)	1 (0.6)
Ankle OA, n (%)	Stage 1		3 (7.5)	3 (1.8)
	Stage 2		1 (2.5)	1 (0.6)
	Stage 3		3 (7.5)	3 (1.8)
	Stage 4		1 (2.5)	1 (0.6)
Joint pain score^d^, n (%)	Mean ± SD	6.0 ± 1.1	5.7 ± 1.3	5.9 ± 1.2
	< 5	15 (11.9)	12 (30.0)	27 (16.3)
	≥5–7	71 (56.3)	19 (47.5)	90 (54.2)
	≥7	40 (31.7)	9 (22.5)	49 (29.5)

*SD* standard deviation, *BMI* body mass index, *OA* osteoarthritis, *K-L* Kellgren and Lawrence

^a^This assessment was conducted for patients with secondary OA

^b^Percentages are based on the number of patients with secondary OA

^c^In knee, shoulder, and elbow joints, Stage of OA is indicated by K-L grading score. In hip and ankle joints, Stage of OA is indicated by stage of each joint

^d^For the 0 to 10 numerical rating scale for pain intensity, 0 indicates no pain and 10 indicates worst pain

### Safety

All TEAEs were experienced by 75.9% (126/166) of patients: 74.6% (94/126) of patients with knee OA and 80.0% (32/40) of patients with other OA. Common TEAEs (≥5%) were nasopharyngitis, eczema, osteoarthritis, injection site joint pain, and contusion (Table 2). Osteoarthritis was regarded as a TEAE when there was worsening of OA at the target joint (1/13 of patients) and developing or worsening of OA at non-target joints (12/13 of patients). Treatment-related TEAEs were experienced by 9.0% (15/166) of patients, 7.1% (9/126) of patients with knee OA, and 15.0% (6/40) of patients with other OA. Treatment-related TEAEs occurred in 3.0% (5/166) of patients after the first injection, 3.0% (5/165) after the second injection, 3.7% (6/163) after the third injection, 5.0% (8/160) during the fourth and seventh injections, and 5.7% (9/158) during the seventh and thirteenth injections. The incidence rate of treatment-related TEAEs was not associated with the treatment period.

**Table 2:** Treatment-emergent adverse events in the safety set

	TEAEs			Treatment-related TEAEs		
	Knee joint	Other joint	Total	Knee joint	Other joint	Total
	*N* = 126	*N* = 40	*N* = 166	*N* = 126	*N* = 40	*N* = 166
Any events	94 (74.6)	32 (80.0)	126 (75.9)	9 (7.1)	6 (15.0)	15 (9.0)
Common events (≥5%)						
Nasopharyngitis	30 (23.8)	11 (27.5)	41 (24.7)	0	0	0
Eczema	6 (4.8)	4 (10)	10 (6.0)	0	0	0
Osteoarthritis	11 (8.7)	2 (5.0)	13 (7.8)	0	0	0
Injection site joint pain	9 (7.1)	5 (12.5)	14 (8.4)	8 (6.3)	5 (12.5)	13 (7.8)
Contusion	13 (10.3)	4 (10.0)	17 (10.2)	0	0	0
Serious TEAEs	10 (7.9)	0	10 (6.0)	0	0	0
Significant TEAEs^a^	0	1 (2.5)	1 (0.6)	0	1 (2.5)	1 (0.6)
Special interest events						
Events at injection site	12 (9.5)	6 (15.0)	18 (10.8)	8 (6.3)	5 (12.5)	13 (7.8)
Gastrointestinal disorders	2 (1.6)	0	2 (1.2)	0	0	0
Anaphylactic reaction	4 (3.2)	3 (7.5)	7 (4.2)	0	0	0
Hypersensitivity	16 (12.7)	7 (17.5)	23 (13.9)	0	0	0

TEAEs were coded using the Medical Dictionary for Regulatory Activities (MedDRA) ver.22.0. OA = osteoarthritis; knee joint = patients with knee OA; Other joint = patients with OA at joints other than knee; NSAIDs =  Nonsteroidal anti-inflammatory drugs; total = whole patients treated concomitantly with or without systemic NSAIDs

^a^Treatment-emergent adverse events (TEAEs) leading to study drug withdrawal are defined as significant TEAEs

No deaths were reported in this study. Twelve serious TEAEs were reported in 10 patients with knee OA. Among them, breast cancer, cerebral infarction, acute myocardial infarction, angina unstable (two events in one patient), and anal polyp were severe. Campylobacter infection, cellulitis (both events in one patient), and appendicitis (*n* = 2) were moderate. Atrial fibrillation and large intestine polyp were mild. Outcomes of all cases were resolved except for breast cancer. The causal relationship to DF-HA was concluded to be unrelated in all cases. One case of treatment-related TEAE associated with palpitations that led to study drug withdrawal was reported in one patient with hip joint OA (0.6%) but it was mild and the patient recovered on the same day without treatment (Table 2).

Regarding TEAEs of special interest, treatment-related joint pain at the injection site was reported in 7.8% of patients. TEAEs associated with GI disorders (gastric ulcer or gastritis erosive), hypersensitivity (e.g., eczema and stomatitis), and anaphylactic reaction (e.g., erythema and urticaria) were observed, but all TEAEs were judged to have no causal relationship to DF-HA (Table 2). For these events of special interest, no serious TEAEs and TEAEs leading to study drug withdrawal or clinically significant events were observed.

For the subgroup analysis of concomitant medication use, the incidence of TEAEs was 63.9% (46/72) in patients who did not receive NSAIDs compared with 85.1% (80/94) in patients treated concomitantly with NSAIDs, in whom system organ classes “infections and infestations”, “musculoskeletal and connective tissue disorders”, and “injury, poisoning and procedural complications” were commonly reported. Incidences of GI disorders were 1.4% (1/72) and 1.1% (1/94) in patients treated concomitantly without and with NSAIDs, respectively. Similarly, TEAEs including events at the injection site were not increased in patients receiving IA injection of HA concomitantly with DF-HA over 24 weeks compared with those who received treatment for 24 weeks or less (Table 3).

**Table 3:** Subgroup analysis of TEAEs by concomitant medication

	N	TEAEs	Treatment-related TEAEs	Gastrointestinal disorders^a^
Without NSAIDs	72	46 (63.9%)	5 (6.9%)	1 (1.4%)
With NSAIDs	94	80 (85.1%)	10 (10.6%)	1 (1.1%)
	N	TEAEs	Treatment-related TEAEs	Events at injection site
Without HA	112	83 (74.1%)	12 (10.7%)	14 (12.5%)
With HA for ≤24 weeks	30	23 (76.7%)	2 (6.7%)	1 (3.3%)
With HA for > 24 weeks	24	20 (83.3%)	1 (4.2%)	3 (12.5%)

*TEAEs* treatment-emergent adverse events, *HA* hyaluronan, *NSAIDs* nonsteroidal anti-inflammatory drugs

^a^Standardised MedDRA Queries (Broad Scope)

For target joint examination, percentages of patients that had worsened joint effusion, swelling, redness, or warmth compared with baseline were in the ranges of 1.3–4.2%, 1.3–3.8%, 0, and 0%–0.6%, respectively, and percentages were not associated with injection times. All events assessed as worsened were judged to not be TEAEs. Regarding X-ray imaging, fractions of patients with structural worsening were 0.6% (1/166), 2.4% (4/166), 1.8% (3/166), and 0% (0/166) for osteophytes, joint space narrowing, osteosclerosis, and deformity of epiphysis at week 52 or last assessment, respectively. One finding of narrow joint space in a patient with knee OA was judged to be TEAE associated with OA at week 24. A causal relationship to treatment in this case was determined to be unrelated and DF-HA was injected through to week 52, when further changes were not found. All other findings were judged not to be TEAEs. There were no noteworthy changes from the baseline laboratory values and vital signs.

### Efficacy

**Fig. 2:**
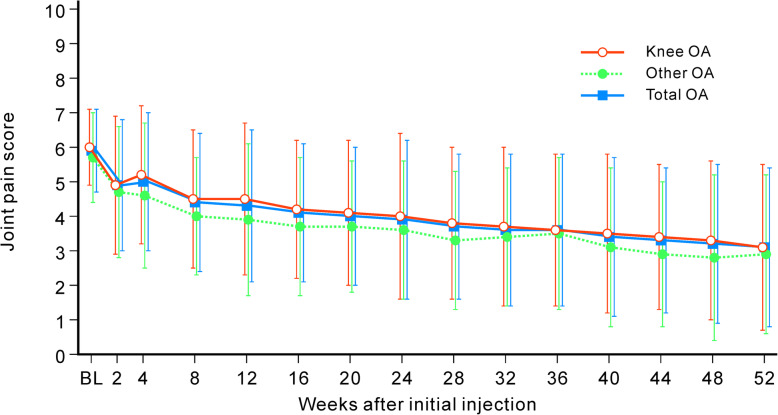
Time series of mean pain scores. Changes in mean pain scores from baseline to 52 weeks. Error bar indicates standard deviation. Total OA = OA including knee-, shoulder-, elbow-, hip-, or ankle-joint OA; other OA = OA other than in the knee joint

The mean joint pain scores (± standard deviation) were 5.9 ± 1.2, 4.9 ± 1.9, and 3.1 ± 2.3 at baseline and at weeks 2 and 52, respectively. Improvement of pain score was observed after the first injection and was maintained until week 52 (Fig. 2). Additionally, similar improvements were noted for the physician global assessment, SF-36 Physical Component Summary, and EQ-5D scores. The proportion of improved patients after each injection was 68.8% (108/157) at week 52 (Table 4).

**Table 4:** Summary statistics of efficacy outcomes at baseline, weeks 2, 12, 24, and 52

	Baseline Mean ± SD	Week 2 Mean ± SD	Week 12 Mean ± SD	Week 24 Mean ± SD	Week 52 Mean ± SD
	*N* = 166	*N* = 166	*N* = 162	*N* = 158	*N* = 157
Pain score	5.9 ± 1.2	4.9 ± 1.9	4.3 ± 2.2	3.9 ± 2.3	3.1 ± 2.3
Patient global assessment (mm)	54.8 ± 19.3	43.4 ± 21.7	36.3 ± 23.7	33.0 ± 22.7	24.9 ± 23.0
Physician global assessment (mm)	54.2 ± 15.5	41.6 ± 19.8	32.8 ± 20.7	31.2 ± 22.1	23.7 ± 21.6
SF-36^a^					
MCS	53.0 ± 8.4	NA	53.1 ± 9.5	53.7 ± 9.4	52.8 ± 9.2
RCS	47.5 ± 13.3	NA	49.0 ± 11.7	49.8 ± 12.1	47.9 ± 12.5
PCS	31.9 ± 11.1	NA	33.0 ± 12.1	33.9 ± 13.0	36.3 ± 12.4
EQ-5D^a^					
QOL score	0.73 ± 0.13	NA	0.77 ± 0.13	0.78 ± 0.14	0.78 ± 0.14
VAS score	68.4 ± 15.6	NA	68.8 ± 18.0	70.9 ± 18.3	71.1 ± 18.6
Responder^b^, n (%)	NA	49 (29.5)	76 (46.9)	91 (57.6)	108 (68.8)

*SF-36* Medical Outcomes Study Short-Form 36-Item Health Survey, *MCS* mental component summary, *NA* not applicable, *RCS* role/social component summary, *PCS* physical component summary, *EQ-5D* EuroQol 5 dimension, *QOL* quality of life, *VAS* visual analogue scale

^a^Lower score indicates more pain or functional disability, and higher score indicates less pain or functional disability. QOL assessment was not conducted at week 2

^b^Patients with improvement in joint pain score from baseline of at least 30%

The time course of pain scores showed an improvement that was similar between knee OA and other joint OA (Fig. 2). WOMAC scores as joint specific questionnaires for the knee joints including subscores to assess stiffness and physical function as well as pain showed continuous improvement in all subscores by repeated injection of DF-HA up to week 52 (Table 5). The score of other joints also showed an improved trend using joint specific questionnaires, although a large variance in values was observed.

**Table 5:** Subgroup analyses in the full analysis set

	Baseline Mean ± SD	Week 2 Mean ± SD	Week 12 Mean ± SD	Week 24 Mean ± SD	Week 52 Mean ± SD
Patients with knee OA or other OA					
Knee OA (n)	126	126	122	121	120
Pain score	6.0 ± 1.1	4.9 ± 2.0	4.5 ± 2.2	4.0 ± 2.4	3.1 ± 2.4
WOMAC score (mm)					
Pain	40.9 ± 18.7	33.1 ± 21.2	29.3 ± 22.3	26.7 ± 22.4	21.7 ± 22.1
Stiffness	39.9 ± 25.9	32.2 ± 23.6	30.2 ± 24.5	28.2 ± 24.7	21.8 ± 22.6
Function	40.5 ± 21.7	35.0 ± 23.0	29.4 ± 22.7	27.2 ± 23.7	22.0 ± 22.8
Total	40.5 ± 20.2	34.4 ± 22.0	29.5 ± 22.3	27.2 ± 23.1	21.9 ± 22.3
Other OA (n)	40	40	40	37	37
Pain score	5.7 ± 1.3	4.7 ± 1.9	3.9 ± 2.2	3.6 ± 2.0	2.9 ± 2.3
Patients with or without analgesics for OA					
Use^a^ (n)	135	135	132	129	128
Pain score	5.9 ± 1.2	4.9 ± 1.9	4.3 ± 2.1	4.0 ± 2.2	3.2 ± 2.3
Not used^b^ (n)	31	31	30	29	29
Pain score	5.7 ± 1.2	4.7 ± 2.2	4.3 ± 2.4	3.7 ± 2.5	2.4 ± 2.4

*n* number of patients in the subgroup, *OA* osteoarthritis, *WOMAC* Western Ontario and McMaster University Osteoarthritis 3.1 index

^a^Patients who used analgesics for OA

^b^Patients who did not use analgesics for OA

Regarding the subgroups treated concomitantly with or without analgesics, the main analgesics for the treatment of OA were NSAIDs and the numbers of patients who used topical and systemic NSAIDs at least once were 111 (66.9%) and 72 (43.4%) patients, respectively. The mean joint pain scores in subgroups treated concomitantly with or without analgesics decreased from 5.9 ± 1.2 and 5.7 ± 1.2 at baseline to 4.9 ± 1.9 and 4.7 ± 2.2 at week 2, and then decreased continuously to 3.2 ± 2.3 and 2.4 ± 2.4 at week 52, respectively. Thus, there was no difference in the improvement of joint pain scores in both subgroups with or without analgesics.

## Discussion

We evaluated the safety and efficacy of DF-HA repeated injection every 4 weeks for 1 year in patients with OA. For the safety evaluation, injected joints were evaluated in addition to TEAEs by target joint examination and structural changes because NSAIDs were reported to be deleterious for cartilage [20], and the progression of joint destruction, joint space narrowing, or osteonecrosis was reported in OA patients treated with anti-nerve growth factor antibody or continuous treatment of IA corticosteroids [21, 22]. The incidence of TEAEs did not increase with the repeated injection of DF-HA for 1 year, the pattern and frequency of TEAEs and joint dysfunction were similar to a previous phase 2 study [10] and confirmatory phase 3 study (unpublished results) as well as other studies of IA treatments in OA patients [23]. Regarding TEAE-related anaphylactic reactions and hypersensitivity, significant adverse events such as those observed in the confirmatory phase 3 study were not observed. Of note, clinically significant TEAEs were not reported. For efficacy, an improvement of OA symptoms was observed after the first injection, and this was maintained up to week 52. Results of a confirmatory phase 3 study indicated that IA injection of DF-HA demonstrated a significant improvement of OA symptoms compared with placebo, and the time course of improvement in WOMAC pain score over 24 weeks in this study was similar to that of the confirmatory phase 3 study (unpublished results). Extrapolating from these similar results, the continuous efficacy of DF-HA might last up to 52 weeks. These safety and efficacy profiles of one injection of DF-HA every 4 weeks indicate the suitability of DF-HA for the treatment of patients with OA, a progressive chronic disease requiring a safe and effective long-term treatment.

Concomitant use of analgesics was prohibited in the phase 2 study [10] and confirmatory phase 3 study but permitted in the current study. Therefore, the safety and efficacy profiles of DF-HA were assessed under such conditions, with consideration of its real-world use. Most concomitant analgesics were oral or topical NSAIDs, which are recommended in the guidelines for OA although oral NSAIDs have a risk of GI disorders [3]. The incidence of TEAEs was higher in patients treated concomitantly with NSAIDs than that in those without NSAIDs. However, some patients used NSAIDs for the management of TEAEs such as infection and pain irrelevant to OA, which was considered the cause of the high incidence of TEAEs in this study. In addition, there was no difference in the incidence of GI disorders reflecting major adverse reactions of NSAIDs between patients treated concomitantly with or without NSAIDs. The low systemic exposure to DF released from DF-HA may have contributed to this result. Regarding efficacy, similar changes in pain scores were observed regardless of the combination of analgesics. This suggests that DF-HA is efficacious in patients receiving NSAIDs with the same mechanism of action but different usage. This might be because DF-HA directly releases DF into the painful site of the joint cavity, in addition to the effect of HA. When oral and topical DF was administered to knee OA patients, the mean concentrations of DF were 15.07 and 4.99 ng/g in the synovium, and 16.76 and 1.96 ng/mL in the synovial fluid 12 h after administration, respectively [24]. One dose of DF-HA (30 mg) contains 3.5 mg of DF [8] and the mean synovial fluid volume is approximately 3 mL in the human knee [25]; therefore, DF-HA IA injection might achieve a higher local concentration of DF for a longer period in the knee synovial fluid than its oral or topical administration, though the actual time course of DF concentration in the synovium or synovial fluid after DF-HA administration in OA patients has not been measured. The mean joint pain score (NRS score) was < 4 in patients receiving DF-HA with or without analgesics. An NRS score < 4 indicates a patient with an acceptable symptom state [26] and a clinically significant improvement from baseline. On the basis of these safety and efficacy results of DF-HA concomitant with NSAIDs, DF-HA may be used in combination with oral or topical NSAIDs, or as DF-HA monotherapy.

OA is a degenerative joint disorder that develops in various joints. The treatment options for OA are similar regardless of the affected joint. A typical medical treatment is pain management with analgesics such as NSAIDs. However, IA injection, which is widely used for knee OA patients, is not a common treatment option for other joints, and data of its efficacy are limited [27–32]. One of the aims of this study was to use DF-HA for various OA; therefore, patients with knee, shoulder, elbow, hip, and ankle OA were enrolled to evaluate the safety and efficacy of DF-HA. Although the interpretation of the results is limited because of the small sample size of the other OA than knee groups, similar trends in safety and efficacy were observed between knee and other joints. Additional trials are needed to determine which joint OA is suitable for DF-HA treatment with an acceptable safety and efficacy profile.

This study reports safety and efficacy data for DF-HA injected repeatedly for 1 year, and provides useful information for its practical use to treat patients with OA joints, and its combined use with NSAIDs and HA. However, this study had several limitations: 1) it was difficult to accurately evaluate the safety and efficacy of DF-HA because this study had no control arm and a large placebo effect was reported for IA injection in OA [33]; 2) no restricted concomitant use of analgesics, including NSAIDs, and IA-HA at the same target joint might have affected the results of the study; 3) sample sizes of subgroups of individuals with joint OA other than knee OA, including patients treated with DF-HA in combination with NSAIDs and/or HA, were too low to evaluate the safety and efficacy or identify risk groups; and regarding IA-HA subgroup analysis, it is also a limitation that the analysis was conducted to evaluate only safety in a small number of patients; 4) treatment/observation periods do not necessarily seem to be enough to evaluate the risk of cartilage loss or OA progression, which is a concern in patients receiving long-term treatment with NSAIDs and IA corticosteroids [20, 21]. Therefore, a well-controlled study conducted over a longer period is required to evaluate the efficacy and safety of DF-HA when employed as a long-term IA-injectable drug. In addition, it will be necessary to evaluate the effect of the difference in patient demographic characteristics, such as K-L grade, on the efficacy and safety of DF-HA to address future clinical questions.

## Conclusions

In the present study, repeated IA injection of DF-HA every 4 weeks for 1 year was tolerated well with no clinically significant adverse events and was suggested to lead to the long-term improvement of OA symptoms. DF-HA may be a useful treatment for patients with OA.

## Data Availability

The datasets generated and analysed during the current study are not publicly available due to confidential nature of the material, but are available from the corresponding author on reasonable request.

## Abbreviations

BMI: Body mass index
C-IRB: Central Institutional Review Board
DF-HA: Diclofenac etalhyaluronate
EQ-5D: EuroQol 5 dimension
FAS: Full analysis set
GI: Gastrointestinal
HA: Hyaluronan
IA: Intra-articular
ICH: International Conference on Harmonization
K-L: Kellgren and Lawrence
MCS: Mental component summary
MedDRA: Medical Dictionary for Regulatory Activities
NA: Not applicable
NRS: Numerical rating scale
NSAIDs: Nonsteroidal anti-inflammatory drugs
OA: Osteoarthritis
PCS: Physical component summary
PREE-J: Patient-Rated Elbow Evaluation-Japanese Version
QOL: Quality of life
RCS: Role/social component summary
SAFE-Q: Self-Administered Foot Evaluation Questionnaire
SD: Standard deviation
SF-36: Medical Outcomes Study 36-Item Short-Form Health Survey
TEAEs: Treatment-emergent adverse events
VAS: Visual analogue scale
WOMAC: Western Ontario and McMaster University Osteoarthritis 3.1 index
